# Genomic variation of human microRNAs and its association with functional features

**DOI:** 10.1007/s00018-025-05936-x

**Published:** 2025-11-19

**Authors:** Mert Cihan, Miguel A. Andrade-Navarro, Enrique Morett

**Affiliations:** 1 Institute of Organismic and Molecular Evolution, Faculty of Biology, Johannes Gutenberg University Mainz, Hanns-Dieter-Hüsch-Weg 15, Mainz, 55128 Germany; 2 Departamento de Ingeniería Celular y Biocatálisis, Instituto de Biotecnología, Universidad Nacional Autónoma de México (UNAM), Av. Universidad 2001, Cuernavaca, Morelos 62210 Mexico

**Keywords:** Non-coding RNA, Conservation, Genetic variants, Population genetics, Target regulation, Co-evolution

## Abstract

Current methods for annotating microRNAs (miRNAs) often rely on phylogenetic conservation or expression data, with less attention paid to the impact of human genetic variation. This limits our understanding of how variation shapes miRNA function and regulatory dynamics across populations. In this study, we systematically annotated genomic variants within human miRNAs and investigated their relationship to functional features and evolutionary constraint. To facilitate this, we developed a population-based conservation metric that integrates allele frequency and positional coverage across miRNA loci. We show that miRNA conservation effectively links to functional roles, as evidenced by associations with higher expression levels, broader target gene regulation, and enrichment in essential biological pathways. Conserved miRNAs also preferentially target genes with fewer alternative polyadenylation sites, indicating more stable and consistent regulatory interactions. This trend is also reflected in miRNAs with both 5p and 3p arms, where the more conserved arm typically regulates more targets, especially when conservation differences between arms are pronounced. Moreover, we find that miRNA genomic variants display population-specific patterns, often co-occurring with target site variants to form compensatory pairs that preserve base-pairing. These events suggest co-evolution and several involve pathogenic variants, indicating that some deleterious regulatory disruptions in target genes may be mitigated through compensatory changes in miRNAs that restore binding.

**Supplementary Information:**

The online version contains supplementary material available at 10.1007/s00018-025-05936-x.

## Introduction

Genomic variability, driven by changes in both protein-coding and non-coding regions, governs key biological processes that influence cellular functions and shape phenotypic diversity across populations [1]. Specifically, variability in non-coding regions significantly influences gene expression by affecting chromatin accessibility, altering mRNA stability, affecting transcription and translation, and modulating key post-transcriptional mechanisms [2, 3]. Among these mechanisms, microRNAs (miRNAs)—small non-coding RNAs—emerge as a critical layer of regulation by binding to complementary sequences on target mRNAs, thereby directing their degradation or repressing their translation [4].

While the initial detection and annotation of miRNAs were primarily guided by criteria such as phylogenetic conservation of sequence and hairpin structure [5], more recent efforts have integrated large-scale small RNA sequencing data to address the complexity of miRNAs. These advances have introduced criteria such as precise read mapping profiles, consistency of mature miRNA 5′ ends, and duplex characteristics, which allow for higher precision in the identification of species-specific miRNAs alongside conserved ones [6]. Challenges in miRNA identification persist due to biases from RNA sequencing quality and depth, leading to misannotation [7]. Additionally, inconsistencies arise from the lack of standardized identification criteria across species, as thresholds for conservation and sequencing read counts vary, complicating accurate identification and comparisons of lineage-specific miRNAs [8]. Moreover, species-specific miRNAs with low conservation and low read profiles are particularly prone to be identified as false positives, necessitating rigorous experimental confirmation to validate computational predictions [6].

While accurate annotation of miRNAs is critical to avoid false positives, the outcome of miRNA function are further shaped by sequence variability in the population. These genetic variants can occur both within the miRNA itself or their target sites, altering seed specificity and therefore target recognition, by disrupting or creating new binding regions in mRNA 3′UTRs [9, 10]. Consequently, variants can lead to changes in gene expression patterns, contributing to phenotypic diversity and disease, with roles in oncogenesis, metabolic disorders, and inflammatory conditions through the modulation of regulatory networks and protein levels [11].

The genomic variability of miRNAs within human populations highlights a clear distinction between high-confidence miRNAs, which show minimal genetic variation likely due to their essential regulatory roles, and species-specific miRNAs, which exhibit greater variability as they adapt to lineage-specific functions [12].

With the availability of extensive human genomic variation data from gnomAD v4.1 [13], we aim to investigate miRNA conservation within human populations by developing a scoring method that evaluates the authenticity and functional relevance of miRNAs. This scoring approach reflects their biological relevance by assessing variant frequency and distribution in relation to their location within the mature miRNA. We further expanded these analyses by examining multiple factors, including target interaction dynamics, and genomic variation characteristics. These include aspects such as annotating target genes, functional enrichment, alternative polyadenylation patterns, co-evolution with targets, and variant properties such as transition-transversion and InDels ratios. Our method aims to enhance our understanding of miRNA function in a context of genomic variant distribution and points towards potential misannotations of miRNAs.

## Methods

### Data collection and processing

We obtained genomic variation data from gnomAD v4.0 [13], which includes 76,215 Whole Genome Sequencing (WGS) and 730,947 Whole Exome Sequencing (WES) datasets. We filtered these datasets to retain only genomic variants corresponding to alleles located within the genomic regions of 2,883 mature human miRNAs and 1,918 precursor miRNAs, as defined by miRBase [6], considering only alleles that passed quality control for genomes. The seed region of each miRNA was defined as positions 2–7 within the mature miRNA sequence. Joint allele frequencies (AF) from gnomAD [13], selected for presence in WGS data but optionally in WES variant data without discrepant AF, were used for downstream analyses. For 156 mature miRNAs there are no reported genomic variants that pass the filters. We consider these miRNAs as having a conservation score of 1 but excluded them from downstream analyses, as they do not provide any population-specific information.

### Conservation score computation

To quantify conservation for each miRNA, we computed an Overall Conservation Score (OCS). This score integrates parameters that capture conservation within the seed region, outside the seed region, the number of positions with variants, and the total number of variants across the miRNA. The Seed Conservation Score (SCS) is defined as

$SCS=1-\frac{\sum \left({f}_{s}\right)}{{n}_{s}}$ where f_s_ represents the AF of a seed variant and n_s_ is the total number of seed variants. Similarly, the Non-Seed Conservation Score (NSCS) quantifies conservation outside the seed region, using the same formula but applied to non-seed variants, defined as

$NSCS=1-\frac{\sum \left({f}_{ns}\right)}{{n}_{ns}}$ where f_ns_ represents the AF of a non-seed variant and n_ns_ is the total number of non-seed variants. The Positional Coverage Score (PCS) evaluates the proportion of variant-free positions within the miRNA and is defined as

$$PCS=1-\frac{{p}_{v}}{L}$$

where p_v_ is the number of unique positions with variants and L is the total length of the miRNA. The Total Variants Score (TVS) assesses the overall burden of variation and is defined as

$$TVS=1-\frac{{v}_{t}}{L}$$

where v_t_ is the total number of variants across the miRNA and L is the total length of the miRNA. The OCS combines these scores with weights assigned to SCS, NSCS, PCS, and TVS, respectively:

$$OCS=\left(\alpha *SCS\right)+\left(\beta *NSCS\right)+\left(\gamma *PCS\right)+\left(\delta *TVS\right)$$

These parameters capture conservation in the seed region, outside the seed region, positional coverage, and the overall burden of genomic variation, respectively.

To determine the optimal weights, we used the expression-conservation overlap as a robust estimation of the functional relevance of miRNAs. Specifically, we aim to maximize the overlap between the most expressed and most conserved miRNAs for various weight combinations. The tested weight combinations are constrained to sum up to 1, with the additional conditions that the weight for the seed region (α) must be higher than the weight for the non-seed region (β) and that each weight must be greater than 0, varying in increments of 0.1.

To ensure a robust evaluation, we consider two independent datasets reporting miRNA expression: the miRNATissueAtlas2 [14] and the Adult Genotype-Tissue Expression (GTEx) database [15]. For each weight combination, we tested thresholds of 5%, 10%, 15%, 20%, and 25% to capture the computed overlaps of the high conserved and top-expressed miRNAs. Although certain miRNAs may be conditionally expressed, those consistently detected across tissues are likely to be genuine and functionally relevant. We therefore used these subsets to find the weight parameters. At the same time, it does not exclude conditionally expressed miRNAs from being identified as highly conserved and such miRNAs can still receive high OCS values based on their variant profiles, regardless of their expression levels.

For each weight combination and threshold, we calculate the average F1-score across the two datasets, integrating both precision and recall to evaluate conservation-expression overlap. To identify the most robust weight combination, we computed the z-score of the average F1-score for each weight combination relative to all others at each threshold. The z-scores quantify how much a weight combination deviates from the mean performance at a given threshold. Finally, we calculated the average absolute z-score across all thresholds for each weight combination. The weight combination with the highest average absolute z-score is selected as the optimal choice, as it consistently outperforms others relative to the mean, highlighting its robust performance across diverse biological contexts. Sequences identical across multiple genomic loci were collapsed under a single mature miRNA name, and the final OCS was computed at the level of mature miRNA sequences.

### miRNA expression

We obtained a comprehensive dataset of 42,494 RPM-normalized miRNA expression values across multiple tissues and conditions from isomiRdb [16], and compared OCS values to the mean expression across the entire dataset. Additionally, tissue average expression values for 2,656 miRNAs were obtained from miRNATissueAtlas 2 [14]. The small RNA-sequencing data from the GTEx Portal used for the analyses described comprises 2,564 miRNAs and were obtained on December 16 2024 [17]. While GTEx and miRNATissueAtlas databases were utilized during the weight optimization process, isomiRdb expression data served as the primary dataset for downstream analyses.

### miRNA targets

Human miRNA targets based on the hg38 human reference genome were downloaded from the microT database [18]. Only binding sites within the 3'UTR regions of genes were considered, and interactions were restricted to those with a default gene-miRNA interaction score of at least 0.7 and a miRNA recognition element (mre) score of 0.01. This resulted in 11,421,667 annotated miRNA binding sites for 183,257 unique miRNA-gene pairs. In addition, we downloaded all conserved miRNA binding sites from TargetScanHuman 8.0 [19] and cross-validated our findings by comparing the number of targeted genes across high- and low-conservation miRNA groups with respect to the presence of APA sites.

### Polyadenylation sites

Polyadenylation sites mapped on hg38 were obtained from PolyASite 2.0 [20]. Only polyadenylation sites located in exon regions of protein-coding genes were considered in the analysis. The representative alternative polyadenylation (APA) site for each gene was defined in PolyASite 2.0 as the position with the highest read support among all APA sites.

### Computation of compensatory variant pairs

Genomic variation in miRNA seed regions or in their binding sites in target genes can disrupt regulatory interactions. We aim to investigate whether variations in miRNA seed regions and their corresponding binding positions exhibit potential co-evolution patterns, ensuring that despite sequence variation, the binding pair remains preserved.

To do this, we obtained 1,851,543 unique miRNA-gene pairs with experimental support from miRTarBase [21] and TarBase-v9 [22]. We then retrieved 3' UTR sequences from the hg38 reference genome using BioMart [23], selecting the longest 3' UTR as the representative sequence. Next, we aligned miRNA seed sequences from miRBase [6] to these 3' UTR sequences, identifying all matching 6mer, 7mer-A1, 7mer-m8 and 8mer miRNA binding sites [24].

By definition, this alignment guarantees at minimum a perfect match between positions 2–7 of the miRNA seed and the target site (6mer). A genomic variant occurring in either the miRNA or the target gene alone would always introduce a mismatch, potentially disrupting the binding. However, when simultaneous variants occur at aligned positions in both the miRNA seed and its 3' UTR binding site, their effect depends on whether they maintain or disrupt base-pairing. If the variants preserve complementary base-pairing, they are classified as compensatory variant pairs, suggesting potential co-evolution. In contrast, if complementary base-pairing is not preserved, they are considered disruptive and likely interfere with miRNA-target regulation. We identified 66,240 compensatory variant pairs, indicating a potential evolutionary relationship between miRNAs and their targets (Supplementary Table 2).

To assess whether these compensatory variant pairs are population-specific, we analyzed AF data from gnomAD, which reports the AF for each variant and each population. A compensatory variant pair was classified as population-specific if both variants reported their highest AF within the same population rather than in any other population. Using this criterion, we identified 14,869 population-specific compensatory variant pairs, while the remaining 51,371 compensatory variant pairs did not exhibit this population-specific pattern.

### Functional enrichment and disease association of miRNAs

To assess the functional relevance of miRNAs, we performed enrichment analysis using MiEAA 2.0 [25], accessed via rbioapi [26], on the top 5% highest conserved (HC) and 5% least conserved (LC) miRNAs based on OCS; each group corresponding to 124 mature miRNAs. The enrichment profiles of over- and under-represented terms were compared to identify patterns of functional divergence and overlap. Categories analyzed included GO terms, pathways, and disease associations and cover only significant terms, based on adjusted p-values. Clinical significance was assessed by cross-referencing variant positions with the ClinVar database [27].

## Results

In this study, we investigated genomic variants within miRNAs and their target sites obtained from gnomAD v4 across different ancestry groups in the human population. To quantify miRNA conservation, we applied a scoring method that integrates AF and positional impact of variants within seed and non-seed regions. We further evaluated this conservation score by comparing HC- and LC-miRNAs (highest and least conserved miRNAs, respectively) in terms of their expression profiles, targeting dynamics, and functional roles. By analyzing allele types and frequencies across ancestry groups, we identified population-specific variants and assessed their potential functional impact. Rare and common variants were characterized to explore their relevance in regulatory processes and potential links to disease. This comprehensive analysis reveals how genomic variation shapes the regulatory roles of miRNAs across human populations, providing insights into their functional significance.

### Characterization of miRNA variants

**Fig. 1:**
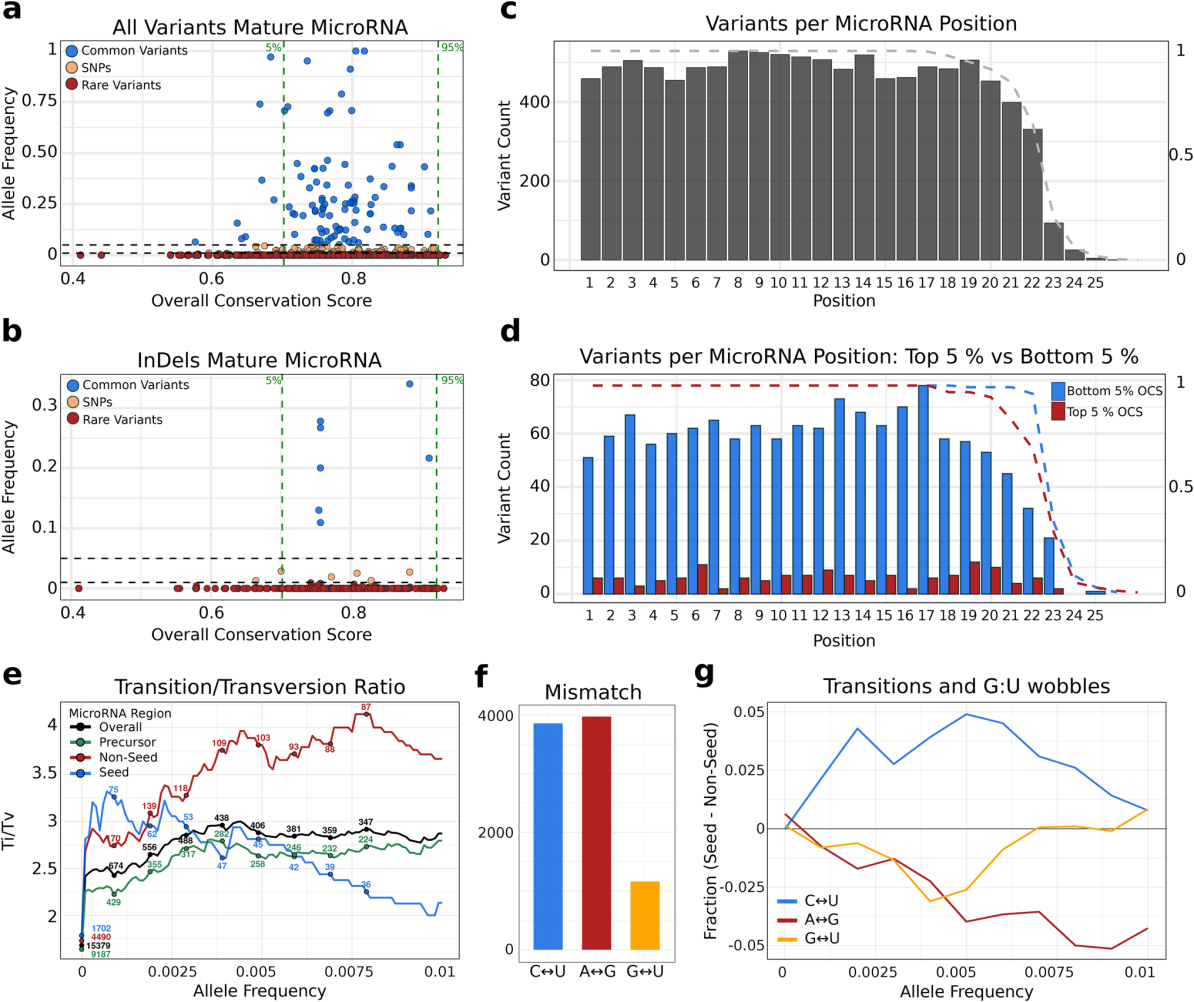
MiRNA Variant Distribution and Conservation in Human Populations. **a** Distribution of AF for all genetic variants in mature miRNA regions. Variants are categorized based on AF thresholds: common variants (AF > 0.05), SNPs (0.01 < AF < = 0.05), and rare variants (AF < = 0.01). The 5% and 95% quantiles for miRNA OCS are indicated. **b** Distribution of insertions and deletions (InDels) in mature miRNA regions, shown with Overall Conservation Scores. Variants are colored according to AF as in **a**. **c** Variant count per position in mature miRNA regions. The dotted line indicates the fraction of mature miRNAs covering a given position length. **d** Same plot as **c** but for the top 5% most conserved and least conserved mature miRNAs (124 in each category). **e** Transition to transversion ratios of AF across different regions: all variants, precursor regions (excluding mature miRNAs), non-seed regions and seed regions of mature miRNAs. The number of variants analyzed in each category is labeled. **f** Barplot showing the total number of transition types and G:U wobbles in mature miRNAs. **g** Seed–Non-Seed differences in mismatch type frequencies across allele-frequency thresholds. Positive values indicate higher fractions in seed regions and negative values indicate higher fractions in non-seed regions

We identified 55,796 miRNA variants, of which 32,713 mapped in precursor miRNAs and 23,083 in mature miRNA regions. Among the mature miRNA variants, 16,707 were located in the non-seed region, while 6,376 were in the seed region. These variants were derived from WGS and WES data. Because many miRNAs are intronic and therefore not consistently captured by exome sequencing, discrepancies between WGS- and WES-derived variant counts are expected [28]. After filtering for variants detected in WGS, the total number of variants was reduced to 27,261, of which 10,677 mapped to mature miRNAs: 7,765 in the non-seed region and 2,912 in the seed region (Table 1). On average, one genomic variant occurs every 2.73 base pairs (WGS: 1 SNV every 5.55 bp), with no significant differences between miRNA regions (precursor: 2.83 bp, non-seed: 2.58 bp, seed: 2.59 bp). We categorized variants by their AF as common (AF > 0.05), single nucleotide polymorphisms (SNPs; 0.01 < AF < = 0.05) or rare (AF < = 0.01). Among mature miRNAs, we found 10,511 rare variants, 98 SNPs, and 68 common variants. Regarding SNPs, precursor miRNAs exhibited a significantly higher SNP density than mature miRNAs (Fisher’s Exact Test, *p* = 0.006). and notably, no SNPs were detected in the HC-miRNAs (Fig. 1a). Within precursor miRNA regions, we identified 32,378 rare variants, 127 SNPs, and 208 common variants from WGS and WES data. Region-specific mapping revealed 7,701 variants in the 5′ lower stem (1 variant every 2.82 bp), 6,313 in the 3′ lower stem (1 variant every 2.99 bp), and 14,152 in the terminal loop (1 variant every 2.48 bp). For hairpins with only one mature strand annotated, we inferred the opposite arm using the canonical 2-nt 3′ overhang to define the loop and lower-stem boundaries [29]. In the flanking regions of precursor miRNAs, we annotated 10,479 variants upstream (1 variant every 3.11 bp) and 10,834 variants downstream (1 variant every 3.01 bp) of the precursor on the primary transcript.

**Table 1:** Genetic variants in miRNAs

Populations	Precursor microRNA			Mature microRNA			Seed		
	Joint	Genomes	Exomes	Joint	Genomes	Exomes	Joint	Genomes	Exomes
African/African American	7,791	6,711	3,171	3,688	3,122	1,496	1,496	1,197	638
Admixed American	5,697	3,556	3,632	2,705	1,580	1,831	1,019	614	690
Ashkenazi Jewish	1,253	731	953	506	301	387	189	115	152
East Asian	3,823	1,989	2,603	1,856	837	1,364	746	349	554
European (Finnish)	2,093	1,074	1652	970	406	804	354	171	293
European (non-Finnish)	19,309	8,395	15,056	9,935	3,878	8,158	3,837	1,457	3,182
Middle Eastern	1,718	591	1,566	817	227	754	290	81	277
South Asian	6,900	2,196	5,967	3,414	926	3,020	1,350	335	1,230
Remaining	5,102	1,678	4,409	2,547	682	2,276	986	259	897
Total	32,713	16,584	24,149	16,707	7,765	13,066	6,376	2,912	4,986

A total of 1,710 variants were identified as insertions or deletions (InDels) (Fig. 1b), with 1,093 occurring in precursor miRNAs, where the InDels density is significantly higher compared to mature miRNA regions (Fisher’s Exact Test, *p* = 0.007). In the mature miRNA regions, 447 InDels were found in non-seed regions, while 170 were located in the seed region. Within mature miRNAs, the distribution of variants remained similar between seed and non-seed regions (Fig. 1c). However, when comparing HC-miRNAs with LC-miRNAs, consistently fewer variants are observed in the highly conserved group. Moreover, within highly conserved miRNAs, positions 7 and 16 showed a notably lower number of variants after accounting for miRNA coverage (Fig. 1d).

Among the annotated variants, we computed the transition-to-transversion (Ti/Tv) ratio across allele frequency thresholds in miRNAs. Seed regions exhibit a higher Ti/Tv ratio than non-seed regions for very rare variants (AF ≤ 0.002), but this trend shifts at higher allele frequencies. Furthermore, precursor regions consistently show lower Ti/Tv ratios than the combined mature miRNA regions. (Fig. 1e). We next examined RNA-level mismatches introduced by transitions. Among mature miRNAs, we found 3,971 pyrimidine transitions (C:U), 3,856 purine transitions (A:G), and 1,163 G:U wobble mismatches (Fig. 1g). Across allele frequency thresholds, A:G transitions were more common in non-seed regions, whereas C:U transitions were enriched in seed regions. G:U wobbles predominated in non-seed regions at low allele frequencies but converged between seed and non-seed regions at higher frequencies (Fig. 1f).

An overview of 55,796 genetic variants classified into distinct regions—precursor, mature, and seed—based on their location within miRNAs, further stratified by sequencing method (genome or exome) and human population groups as annotated by gnomAD database. Variants from genome-sequencing and exome-sequencing can be overlapping.

### Conservation scoring and miRNA confidence analysis

**Fig. 2:**
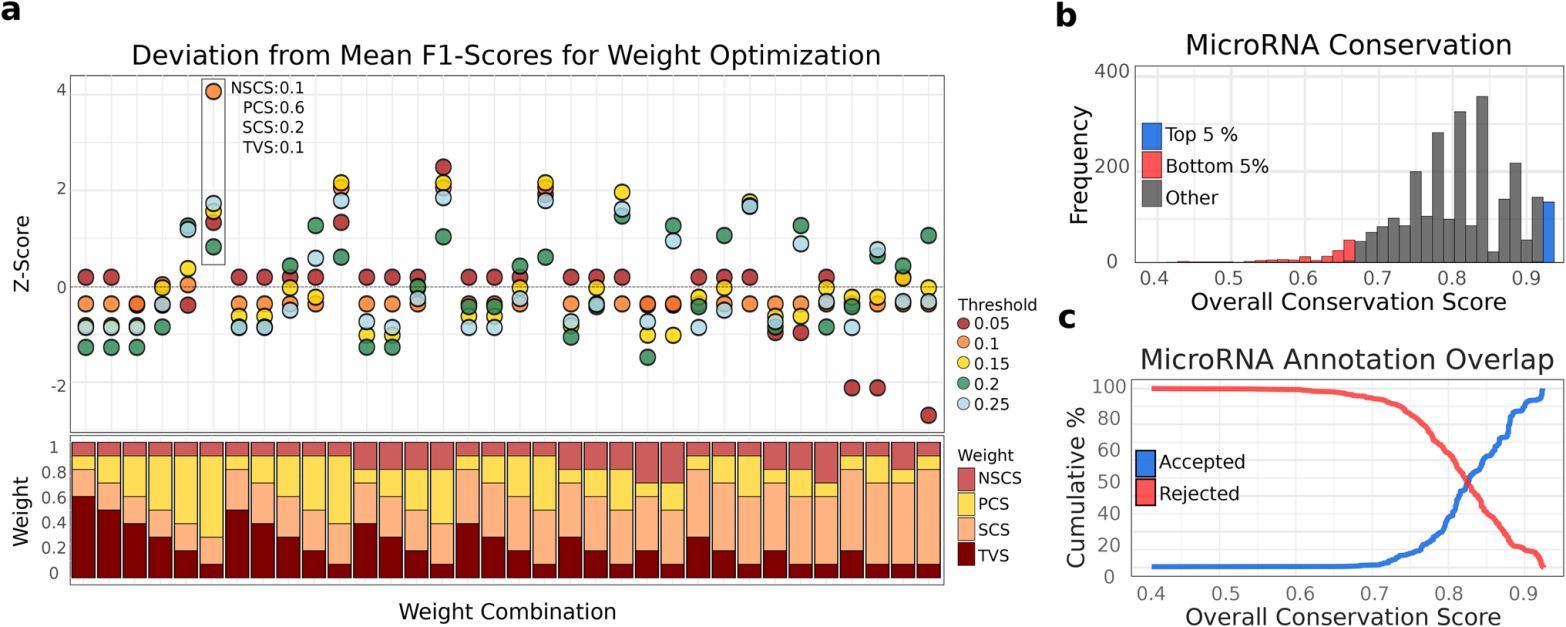
Weight optimization and overall conservation score. **a** Weight Optimization for Seed Conservation Score (SCS), Non-Seed Conservation Score (NSCS), Positional Coverage Score (PCS), and Total Variants Score (TVS). The stacked bar plot at the bottom visualizes the weight combinations for these scores, ensuring the weights sum to 1. For each weight combination, we computed the overlap of conserved miRNAs (based on the computed OCS) with the top-expressed miRNAs derived from the miRNA Tissue Atlas and GTEx expression data, evaluated at different thresholds on the OCS. At each threshold, F1 scores for the overlaps were calculated, and a z-score was computed to indicate the deviation from the average F1 score across thresholds, identifying the optimal weight configuration (see Methods for details). **b** Distribution of OCS across all miRNAs. The top 5% most conserved miRNAs, as defined by OCS values, are highlighted in blue, while the bottom 5% are highlighted in red. **c** Cumulative Coverage of miRNA Confidence by OCS. Line plots illustrate the cumulative percentage of miRNAs from mirGeneDB, categorized by their confidence (accepted; rejected), as covered by OCS

To quantify miRNA conservation from human population variant data, a scoring approach was developed that integrates four key parameters: allele frequency-based conservation in the seed and non-seed regions of mature miRNAs (SCS and NSCS, respectively), positional coverage (PCS), and total number of distinct variants. These four parameters were weighed and combined into an Overall Conservation Score (OCS). We evaluated different weight combinations (34 combinations; see Methods for details) by the overlap between highly scored and highly expressed miRNAs at various thresholds. The hypothesis here was that miRNAs with demonstrated and clear expression are very likely real; conversely, miRNAs that show low or no expression in many samples have high probability of not being real. Importantly, this approach does not exclude conditionally or cell-type–specific miRNAs from being identified as highly conserved, as such miRNAs can still score highly based solely on their variant profiles, independent of their expression levels. The most effective weight combination assigned the highest importance to PCS (0.6), followed by SCS (0.2), NSCS (0.1), and TVS (0.1). This weight combination produced the highest average overlap between highly conserved and highly expressed miRNAs across all thresholds, emphasizing the critical role of positional variant burden in defining conservation. In particular, this weight combination captured the HC-miRNAs better at the threshold of 5%. OCS computation relying mainly on AF performed suboptimally, indicating that it is insufficient for accurately predicting conservation (Fig. 2a).

The second-best weight combination (PCS = 0.4, SCS = 0.3, NSCS = 0.2, TVS = 0.1) outperformed those assigning an even higher weight to PCS (e.g., PCS = 0.5) at higher thresholds. This suggests that while positional conservation is a key factor, increasing its weight beyond a certain point does not necessarily improve performance. Instead, incorporating allele frequency-based conservation parameters, particularly non-seed conservation, enhances predictive accuracy. This effect becomes more pronounced at the tested thresholds, where AF considerations play an increasingly significant role in distinguishing conserved miRNAs (Fig. 2a).

The distribution of conservation scores among mature miRNAs indicates that a subset of miRNAs exhibits particularly low conservation, distinguishing them from the majority. The average conservation score is 0.82, with a standard deviation of 0.07 (Fig. 2b). To further validate the OCS, we compared conservation scores with all curated annotations from MirGeneDB, which defines sets of accepted and rejected human miRNAs [8, 30]. The cumulative distribution analysis reveals a clear trend where miRNAs annotated as high-confidence are predominantly found among the most highly conserved miRNAs according to OCS. Conversely, miRNAs with the lowest conservation scores are more frequently associated with low-confidence annotations in mirGeneDB (Fig. 2c). This pattern supports the agreement between conservation scores and annotation confidence, and therefore reinforces the OCS as an effective score to distinguish functionally relevant miRNAs. In addition, we examined whether paralogous copies of miRNAs influence conservation levels. Among 2,496 mature miRNAs with variant data, 116 are encoded by multiple genomic loci while 2,380 occur as single copies. Multi-copy miRNAs show significantly higher conservation than single-copy miRNAs (Wilcoxon test, *p* < 0.05). A comprehensive list of conservation scores for each mature microRNA, along with their corresponding parameters, is provided in Supplementary Table 1.

### Population-specific miRNA variability

**Fig. 3:**
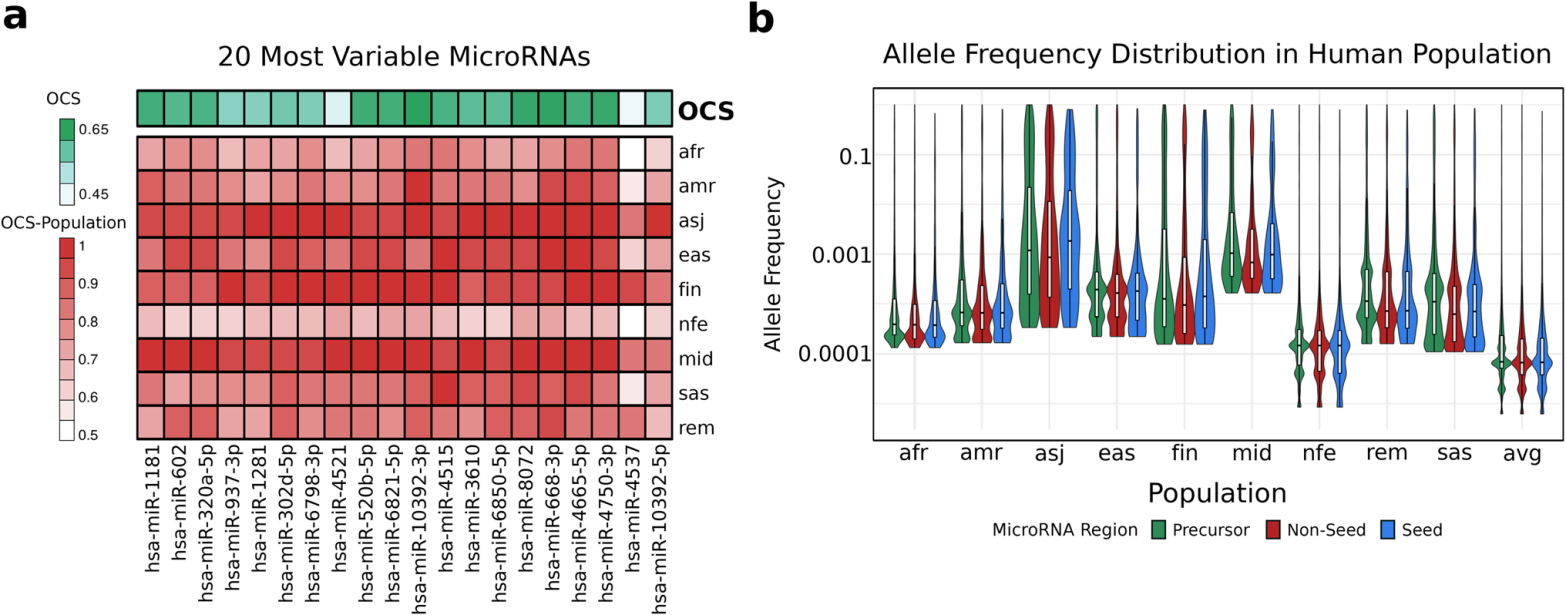
MiRNA variants in human populations. **a** Heatmap of population-specific OCS values, displayed for the 20 most variable miRNAs. The populations considered include African/African American (afr), Admixed American (amr), Ashkenazi Jewish (asj), East Asian (eas), Finnish (fin), Non-Finnish European (nfe), Middle Eastern (mid), South Asian (sas), and Remaining (rem). **b** Allele distribution across human populations shown for precursor miRNA regions (excluding mature miRNAs), non-seed regions, and seed regions of mature miRNAs. The joint AF is presented as an average (avg)

The OCS can be computed using subsets of variants corresponding to populations (as annotated in gnomAD). Population-specific analysis of mature miRNA conservation revealed variability across different groups. For example, hsa-miR-4537, despite having low overall conservation, remains relatively preserved in Finnish, Middle Eastern, and Ashkenazi Jewish populations. Similarly, hsa-miR-10392-5p is notably conserved only in Ashkenazi Jews, suggesting a potential role in population-specific adaptation or genetic drift (Fig. 3a). The population-specific analysis indicated that the conservation trends described above are consistent among populations. For example, we observed that the distribution of allele frequencies across all miRNA regions is significantly different between precursor and mature miRNA regions across all populations, with precursors harboring more alleles at higher frequencies (Mann–Whitney U, *p* < 0.05). No significant differences were observed between seed and non-seed regions, except for the East Asian population (Mann–Whitney U, *p* < 0.05; Fig. 3b).

### Functional impact of miRNA conservation on gene regulation

**Fig. 4:**
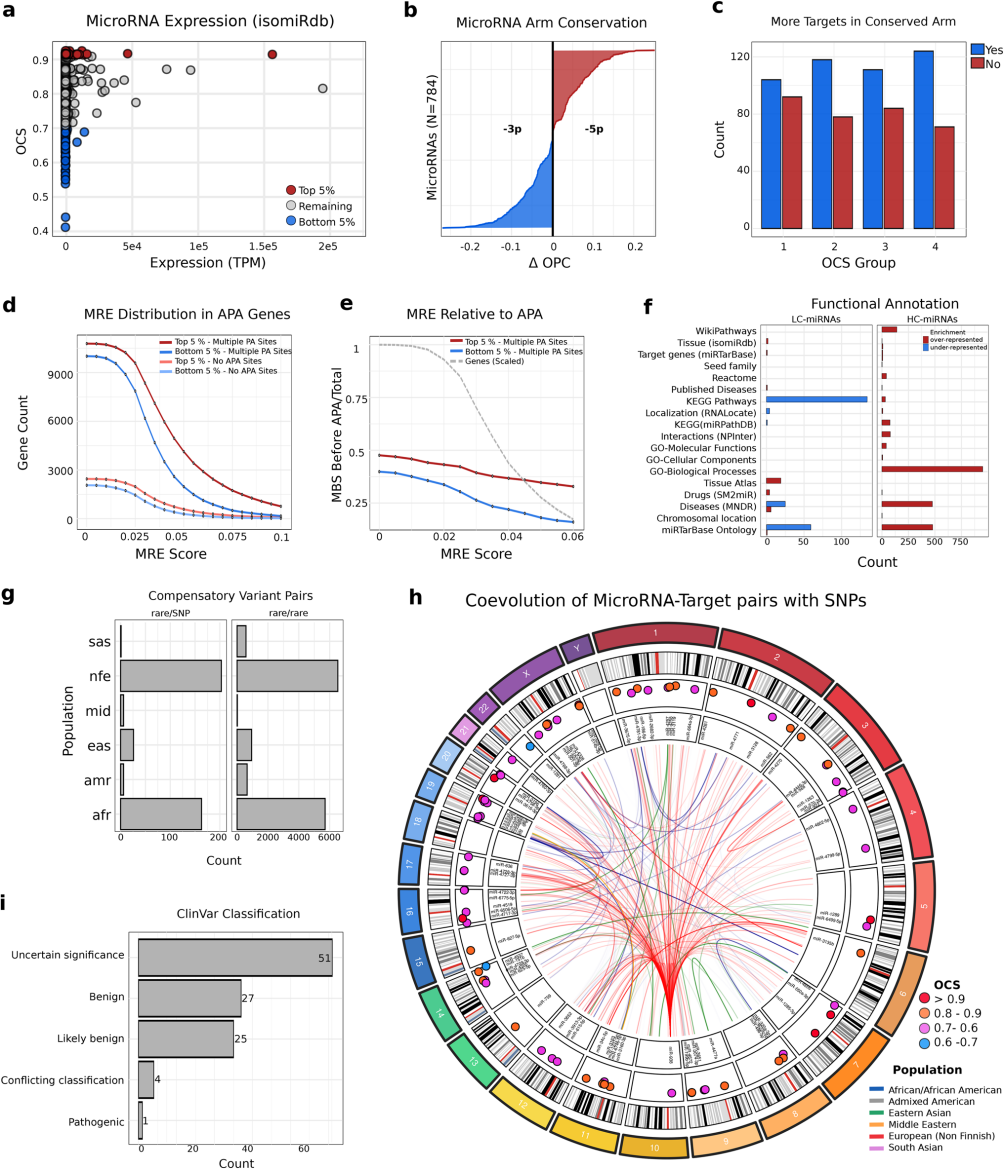
MiRNA Conservation: Expression, Targeting, and APA Dynamics. **a** Normalized mean expression levels of miRNAs from isomiRdb and OCS values. **b** Sorted differences in OCS between the -3p and -5p arms of mature miRNAs. Blue represents cases where the -3p arm is more conserved, while red indicates higher conservation for the -5p arm derived from the same precursor miRNA. **c** miRNAs with two arms were grouped into four bins based on their OCS differences, and the number of cases where the more conserved arm had more target genes was counted. **d** Number of genes lacking APA regulation for top 5% and bottom 5% miRNAs, as well as for genes with APA, across different thresholds of miRNA binding site scores (MRE). **e** Ratio of binding sites before a representative APA site per gene for top 5% conserved and bottom 5% conserved miRNAs. The number of genes considered for each threshold of miRNA binding is scaled and shown in grey. **f** Overview of categories with significantly enriched terms for of HC-miRNAs and LC-miRNAs identified using miEAA (see Methods for details). **g** Counts of co-occurring compensatory variants in miRNA binding sites, with the highest frequency observed in the same population. **h** Circos plot illustrating co-evolved miRNA–target gene variants that are compensatory bopopulation-specific OCS values, displayed for the 20 most variable miRNAs. Theth in the miRNA seed and at its binding site within the human genome. Dots represent the OCS value of the corresponding miRNA. Connections are drawn when both variants share the highest AF for the same population, with increased opacity representing higher AF. At least one of the two variants must be a SNP (0.01 < AF < = 0.05). **i** ClinVar classifications of population-specific compensatory variants

Since our score of miRNA conservation was optimized using expression, it is important to examine how expression levels correspond to this measure to see if we induced any undesired bias. Mean expression levels across diverse biological conditions and tissues from isomiRdb were analyzed in relation to miRNA OCS values. A pattern emerged in which, as expected, lower conservation was consistently linked to reduced expression, but higher conservation did not necessarily imply high expression (Fig. 4a). This indicates that the score can still highlight biologically relevant miRNAs that may otherwise go unnoticed in conventional profiling.

Measuring miRNA expression is inherently linked to its precursor form, where both the 5p and 3p arms are transcribed together and subsequently measured as a single unit. While expression levels of both arms may be correlated, differences in biological activity and functional stability could lead to distinct conservation patterns. To investigate this, we analyzed 784 precursor miRNAs with annotated 5p and 3p arms, comparing their respective OCS values. We found that in 380 cases, the 3p arm is more conserved, while in 404 cases, the 5p arm shows higher conservation. The distribution of conservation differences between the two arms is balanced (Fig. 4b). Permutation analysis of OCS values reveals significantly lower conservation differences between miRNA arms than expected by chance (*p* < 0.01), suggesting a strong tendency for 3p and 5p arms to maintain similar conservation levels. This indicates potential functional or evolutionary constraints preserving balanced conservation between both strands. To assess whether conservation differences between miRNA arms influence their functional relevance, miRNAs were binned based on their OCS difference, and the number of target interactions (as defined in the microT database [18]) for each arm was analyzed. In the group with the smallest conservation differences, the trend remains subtle, yet the more conserved arm tends to have a higher number of target interactions. However, in the group with the largest OCS differences, this pattern becomes more pronounced, with 124 cases compared to 74 cases where the more conserved arm also exhibits a greater number of target genes, suggesting a potential link between conservation and regulatory significance (Fig. 4c). To test if this association exceeds random expectation, a permutation analysis confirmed a significant difference from chance (*p* < 0.01), supporting a link between conservation and miRNA targeting preferences.

Alternative polyadenylation (APA), leading to different length of the 3' UTRs, influences miRNA binding site availability [31]. We tested whether genes lacking APA sites, which maintain stable miRNA binding, are more frequently targeted by highly conserved miRNAs compared to the least conserved ones, integrating OCS scores to assess this relationship. We observe that among 2,784 genes lacking APA sites, the HC-miRNAs consistently target more genes than the LC-miRNAs across multiple thresholds of miRNA binding site scores. For instance, without restriction on binding site scores, this difference is 2,449 vs. 2,061 genes, while considering only binding sites with an mre score >= 0.1, the difference becomes 70 vs. 28 genes. Similarly, among 15,955 genes with multiple APA sites, this trend persists. Without restriction on binding site scores, the HC-miRNAs target 10,784 genes compared to 10,009 for the LC-miRNAs. When considering only binding sites with an MRE score >= 0.1, this difference increases to 760 vs. 166 genes (Fig. 4d).

Further, for each miRNA, we investigated how frequently its miRNA binding sites are located upstream of a representative APA site (on genes with multiple APA sites). We found that this frequency is consistently higher for the most conserved miRNAs (0.48) compared to the least conserved miRNAs (0.39). This difference becomes more pronounced at higher binding site stringency thresholds (Fig. 4e). To validate these results independently, we repeated the analysis using conserved binding sites from TargetScanHuman [19] and confirmed that highly conserved miRNAs preferentially target genes lacking APA sites (911 of 7,339 targets, 12.4%) compared to the least conserved miRNAs (50 of 622 targets, 8.0%; Fisher’s exact test *p* < 0.01). This independent dataset thus supports our conclusion that conserved miRNAs are preferentially associated with stable miRNA regulation.

As an additional measure of OCS validity, we assessed the functional implications of miRNAs and their target genes by annotating and contrasting the Gene Ontology (GO) term enrichment and pathway associations of genes regulated by the HC-miRNAs and LC-miRNAs. This analysis evaluates whether higher conservation corresponds to greater regulatory involvement in essential biological processes and key molecular pathways. We found 2,341 significant terms for HC-miRNAs compared to 257 significant terms for LC-miRNAs. In particular, no significant GO terms were identified for LC-miRNAs, whereas HC-miRNAs were significantly associated with 950 GO biological process terms. Additionally, we observed that significant terms for LC-miRNAs are particularly associated with under-representation in KEGG-pathways and GO terms derived from miRTarBase. The large discrepancy in enriched terms annotated between HC-miRNAs and LC-miRNAs supports the notion that conservation is strongly linked to functional relevance, with HC-miRNAs engaging in more biologically significant and well-annotated regulatory roles (Fig. 4f).

### Evolutionary and clinical insights into compensatory miRNA-target variant pairs

To investigate potential co-evolution patterns between miRNAs and their targets, we analyzed variants occurring in both the miRNA seed region and the corresponding binding site in the 3' UTR of the target gene, while preserving complementary base-pairing.

Interestingly, we identified 66,240 compensatory variant pairs in which sequence variation in both the miRNA and its target maintained base-pairing. Among these, 14,869 pairs exhibited their highest AF in the same population and were classified as population-specific, comprising 14,464 rare compensatory variant pairs and 405 cases in which one or both sites contained a SNP (Fig. 4h; see Methods for details). These compensatory pairs were located in 5,792 6mer, 2,576 7mer-A1, 4,519 7mer-m8, and 1,982 8mer miRNA binding sites. It is noteworthy that compensatory variants can only arise in the context of an initially deleterious mutation that disrupts efficient binding of a miRNA to its target, whether through a change in the miRNA itself or in its binding site. This means that deleterious mutations can sometimes survive in the population and that there is a strong selection pressure to reestablish the regulatory interaction by a compensatory mutation.

The strongest signals of population-specific compensatory variant pairs were observed in the Non-Finnish European population (6,525 rare variants; 203 with one SNP) and the African population (5,702 rare variants; 159 with one SNP; 4 with both SNPs) (Fig. 4g, h). We interpret these rare variants as those that arose recently, in contrast to the frequent ones that have arisen a long time ago, such that they are present in a large proportion of the population. Moreover, the African population harbors a significantly greater proportion of compensatory to disruptive binding pairs compared to all other populations, suggesting stronger evolutionary constraints or selective pressures shaping miRNA-target interactions (Fisher's Exact Test, *p* < 0.01). Significance is retained when the analysis is restricted to binding sites stronger than 6mers (Fisher’s Exact Test, *p* < 0.01). This pattern may also reflect the higher genetic diversity found in African populations [32].

Co-evolution appears to be more frequent in HC-miRNAs than in LC-miRNAs, as HC-miRNAs exhibit a significantly higher ratio of compensatory to disruptive binding pairs at binding positions (Fisher’s Exact Test, *p* < 0.01; consistent for sites stronger than 6mers). This suggests that evolutionary constraints are stronger in functionally essential miRNAs, where compensatory variants may act to preserve regulatory interactions despite sequence variation.

To evaluate the clinical significance of these population-specific compensatory variant pairs, we cross-referenced them with ClinVar and identified 108 matches. Of these, one variant is classified as pathogenic: MMACHC, co-occurring with a compensatory variant in hsa-miR-6516-5p. This compensatory variant pair is associated with methylmalonic aciduria and homocystinuria (7mer-m8 binding site), in the Non-Finnish European population. Moreover, 8 variants have conflicting pathogenicity classifications, and 109 are of uncertain significance (Fig. 4i). While these variants are identified as pathogenic or likely pathogenic, we propose that their effects are through disruption of a miRNA target site and that they can be compensated by the corresponding variation in the interacting miRNA.

To better understand the impact of variants in targeting-dynamics, we also analyzed the remaining 51,371 compensatory variants that did not meet the criterion of population-specificity. Removing this restriction accounts for unequal population representation in the data and natural AF fluctuations that could obscure evolutionary patterns. Additionally, it helps identify functional compensatory mechanisms that persist across multiple populations rather than being confined to a single group. We identified 359 compensatory variant pairs with at least one variant annotated in ClinVar, including 354 in target genes and 5 in miRNAs. Notably, one variant in the COMT target gene, co-occurring with hsa-miR-3907, is classified as a drug response variant associated with Tramadol response. In addition, we observed 8 cases with conflicting pathogenicity classifications and 202 cases of uncertain significance (Supplementary Table 3).

## Discussion

This study investigates the population-specific conservation patterns of miRNAs, emphasizing their variability, functional roles, and evolutionary significance across diverse human populations. By leveraging the expanded gnomAD v4 dataset [13], which provides substantially greater coverage than gnomAD v3 (a fivefold increase in WGS and a sixfold increase in WES datasets), we identified 55,796 variants in precursor and mature miRNAs. This represents an approximately 2.3-fold increase in variant data within genomic miRNA coordinates as compared to prior characterizations [12], highlighting the enhanced resolution and expanded analytical scope provided by the latest genomic resources.

The computation of OCS emphasizes positional conservation as the most impactful determinant of miRNA functionality. While the seed region’s role in determining binding specificity and regulatory activity is well established [4, 33], the non-seed region also plays a critical role by stabilizing 3′-end interactions [19, 34], enabling the recognition of non-canonical target sites, the creation of functionally relevant isomiRs through altered enzymatic cleavage patterns that shift seed location [35] and allowing RNA editing in non-seed regions, which impacts loading efficiency in the RISC complex [36]. This dual importance supports the rationale for scoring variant-free positions equally in both seed and non-seed regions to comprehensively capture the full spectrum of miRNA functionality. The optimization of the computation of the OCS based on expression-conservation overlap implies read coverage as a key metric for miRNA annotation, linking it to greater annotation confidence [37, 38]. This approach is further validated by demonstrating a link between higher expression levels and functional significance in our analyses, reinforcing the role of miRNA expression across multiple biological conditions and tissues in inferring function [25, 39]. Additionally, we validated our findings through comparison with the accepted and rejected miRNAs from mirGeneDB [8, 30].

To further validate OCS computation, comparisons between HC-miRNAs and LC-miRNAs reveal consistent alignment with biological expectations. LC-miRNAs, while detectable, present lower expression levels compared to HC-miRNAs, suggesting their functional roles may be more context-dependent or specialized. Further, these miRNAs with low expression levels have been linked to less connected target networks [40]. In contrast, HC-miRNAs show balanced conservation between -5p and -3p arms, and in cases of imbalance, the less conserved arm consistently targets fewer genes, reinforcing the link between conservation and regulatory relevance. HC-miRNAs also preferentially target sites upstream of representative APA sites. Cell-type-specific 3′UTR shortening correlates with the loss of binding sites for functional microRNAs, implying selective evasion of miRNA regulation [40].Their higher frequency of enriched pathways and Gene Ontology terms further emphasizes their functional significance, whereas LC-miRNAs show sparse associations. Moreover, we find that multi-copy miRNAs are more conserved than single-copy counterparts, consistent with the expectation that redundancy across loci enhances robustness. This finding is counterintuitive since one could have expected that multiple copies of the same miRNA could be more robust to mutation than single copy ones. Patterns of genetic variation also support these findings. The reported density of 1 SNV per 5.55 bp within precursor miRNAs is lower than the genome-wide average of 1 SNV per 4.9 bp [13]. Although no significant differences in SNV density were observed between seed and non-seed regions across all mature miRNAs, a higher density was observed in non-seed regions compared to seed regions when contrasting HC-miRNAs with LC-miRNAs, aligning with the idea that non-seed regions in LC-miRNAs are under reduced evolutionary pressure, allowing for greater variability.

Additionally, precursor miRNAs exhibit a significantly higher density of common variants across all populations, again reflecting their greater tolerance for variation likely due to reduced functional constraints compared to mature miRNA regions [41]. Regional mapping showed slightly higher variant densities in terminal loops compared to lower stems, consistent with the lower stem’s essential role in Drosha processing [42]. While overall Ti/Tv ratios of miRNAs align with previous findings [43], we observed higher Ti/Tv ratios in seed regions for rare alleles, preserving binding stability, while higher-frequency alleles favor non-seed regions, reflecting relaxed selective pressure. Given the absence of SNPs in HC-miRNAs, this pattern underscores the critical role of seed regions in maintaining stable miRNA-mRNA interactions, whereas non-seed regions in LC-miRNAs tolerate greater variability due to reduced constraints. The higher transition rate observed for pyrimidines in seed regions aligns with expectations from RNA structural studies, which show that pyrimidine–pyrimidine mismatches are structurally more compatible with the A-form helices adopted by miRNA precursors and miRNA–mRNA duplexes than bulkier purine–purine mismatches [43, 44].

Collectively, these findings validate the biological relevance of OCS, demonstrating its capacity to distinguish miRNAs with critical regulatory roles from those with limited impact. Moreover, OCS effectively identifies confidently annotated miRNAs and distinguish from the ones that may have lost functionality through evolutionary divergence.

Our analyses identified population-specific differences in conservation of miRNAs, such as in the case of hsa-miR-10392-5p, which is more conserved in Ashkenazi Jews than in other populations. These findings suggest that certain miRNAs may have evolved lineage-specific regulatory roles or adaptations influenced by population-specific selective pressures or genetic drift. Additionally, we observed that certain miRNA variants often co-occur with their corresponding binding site variants in a population-specific manner. The fact that we found many compensatory mutations highlights a very interesting phenomenon of surviving deleterious mutations in a population until they are rescued by a second mutation that restores the base-pairing. Our results show that these mutations occur rather frequently in the African population, which harbors the highest genetic diversity among all populations studied [45]. Among these co-occurring population-specific variants, three cases were identified as likely pathogenic, highlighting their potential clinical relevance. Specifically, variants in target genes MMACHC and COMT that were identified as pathogenic or responding to drugs, are accompanied by compensatory miRNA variants in the respective binding position. These findings highlight the interplay between population-specific genetic diversity and miRNA-mediated regulatory mechanisms, underscoring the potential clinical relevance of such co-evolutionary patterns. With the expanding knowledge of the links between genomic variants and disease, our annotation of potentially co-evolved compensatory variant pairs of miRNAs and target genes offers a valuable resource for investigating these relationships.

We are aware that our study has some limitations: These include the bias introduced by the over-representation of European samples within the gnomAD dataset [13], the discrepancies in AF annotation resulting from differences between WES and WGS data [46], and the lack of sample-specific annotations for co-occurring variants. This last limitation hinders our ability to fully conclude the biological function of co-occurring variants in miRNA seed regions and their corresponding target sites [47, 48].

In conclusion, our study highlights miRNA conservation patterns across human populations using a novel scoring system that integrates genomic variability, further validated with correlations with targeting dynamics and functional enrichment. By distinguishing highly conserved from less conserved miRNAs, we reveal their regulatory significance, population-specific adaptations, and compensatory co-evolutionary patterns.

## Supplementary Information

Below is the link to the electronic supplementary material.

Supplementary file1 This table includes all scoring values for mature miRNAs. Specifically, it presents the SCS, NSCS, PCS, TSV parameters, and resulting OCS values for all mature miRNAs. (XLSX 122 KB)

Supplementary file2 This table presents all population-specific compensatory variant pairs (see Methods for details). The columns include the miRNA and target gene interacting, the chromosome and position of each aligning variant, the population in which both variants have the highest AF compared to other populations, and the type of variant—either both being rare (AF >0.01) or one being a common SNP (0.01 >AF < 0.05) while the other remains rare. (XLSX 7638 KB)

Supplementary file3 This table presents ClinVar records for all compensatory variant pairs in which at least one variant is reported in the ClinVar database. It includes the interaction partner where the variant was found (gene or miRNA), the miRNA name, the gene name, the aligning genomic locations of both, the ClinVar classification, and the list of phenotypes associated with the respective variant. (XLSX 42 KB)

## Data Availability

The code for computing scores and analyses is available at https://github.com/mcihan0bioinf/miriants. Overall conservation scores for microRNAs are provided in the supplementary tables.

## Abbreviations

AF: Allele frequencies
APA: Alternative polyadenylation
GTEx: Adult genotype-tissue expression
GO: Gene ontology
HC: Highest conserved
InDels: Insertions or deletions
LC: Least conserved
miRNAs: MicroRNAs
mre: MiRNA recognition element
NSCS: Non-seed conservation score
OCS: Overall conservation score
PCS: Positional coverage score
SCS: Seed conservation score
SNP: Single nucleotide polymorphisms
Ti/Tv: Transition-to-transversion
TVS: Total variants score
WES: Whole exome sequencing
WGS: Whole genome sequencing
